# Spatial Difference and Equity Analysis for Accessibility to Three-Level Medical Services Based on Actual Medical Behavior in Shaanxi, China

**DOI:** 10.3390/ijerph18010112

**Published:** 2020-12-26

**Authors:** Kan Wang, Jianjun Bai, Xing Dang

**Affiliations:** School of Geography and Tourism, Shaanxi Normal University, Xi’an 710119, China; wangkanstar@snnu.edu.cn (K.W.); D-Star@snnu.edu.cn (X.D.)

**Keywords:** medical services, network analysis, accessibility, equity, Shaanxi province

## Abstract

The contradiction between the supply and demand of public medical resources in China is serious. On the basis of the “graded diagnosis and treatment” model, the Chinese government divides the medical grade and adjusts the allocation of medical facilities so as to alleviate the adverse impact of these issues on residents’ health. Although the government tries to guide residents’ medical treatment according to the level of medical facilities, there are differences between residents’ medical treatment mode and policy rules in reality. Therefore, it is of great significance to explore spatial differences in accessibility to medical services for residents on the basis of the actual medical behavior. This article takes Shaanxi province as the research area, and uses the improved node cost network analysis method with the space-time distance model and the two-step floating catchment area method, respectively, to analyze the spatial differences of accessibility to three-level medical services and evaluate the equity of accessibility in different areas and groups in Shaanxi. Results showed that the overall level of accessibility to primary medical services in the province is good, and spatial distribution is balanced; the polarization of accessibility to secondary and tertiary medical services is a serious issue, and within the research area, a band-shaped multicore spatial structure was formed with the built-up areas of various cities as high-level centers of accessibility. Provincial residents have poor equity to access three-level medical services, and the equity of accessibility to primary medical services is better than that to highly specialized medical services. There is no obvious gap between accessibility to three-level medical services for the aging and the nonaging populations in Shaanxi, but the unfair phenomenon between agricultural and the nonagricultural populations is prominent. In addition, this article found that the improvement in traffic conditions can produce space-time convergence and effectively weaken spatial deprivation. Therefore, developing public transportation is an effective approach to improve the equity of accessibility to medical services.

## 1. Introduction

With economic growth and social development, primary contradictions in China were transformed into contradictions between people’s increasing need for a better life, and unbalanced and inadequate development, of which contradictions in the medical and health field are particularly serious [1]. Since the reform and opening-up, China’s public health rapidly developed, but many social problems were exposed, mainly manifested in the high concentration of medical resources in central cities, which has caused residents in remote area difficulty in seeking medical treatment. At the same time, the total amount of medical resources is insufficient, and residents face strong competition in accessing public medical resources. These problems have caused great harm to the national health. The key to solve the contradiction between supply and demand of medical resources is to analyze the differences of residents’ ability to obtain medical services through their medical behaviors, so as to guide the formulation of relevant policies. At present, the actual medical behavior of Chinese residents is mainly affected by the different medical needs caused by disease types. Therefore, the accurate and reasonable evaluation of the current state of medical services on the basis of actual medical needs of residents to guide policy formulation in order to promote the rational and equitable distribution of medical facilities and maximize social benefits is in line with people’s wishes, which is an important means to maintain economic development and social stability [2,3].

Over the past few decades, research on the allocation of public medical facilities has received extensive attention. Relevant studies usually employ accessibility as an evaluation method to analyze medical resources’ spatial differences. Generally speaking, accessibility indicates how convenient it is for an object to reach a certain location and obtain a certain opportunity through specific traffic conditions. Research mainly focuses on the spatial pattern of accessibility to medical facilities. Wei Hu et al. studied the spatial distribution of medical accessibility of Shenzhen residents under rapid urbanization [4]. On the basis of Baidu map API, Peijun Rong et al. used communities as research units to analyze spatial differences in accessibility to medical facilities for residents in different areas of the city of Zhengzhou, and indicated communities with a lack of medical services through classification [5]. Xiaokun Gu et al. analyzed spatial differences in accessibility to medical resources in Shanghai [6], and provided references for research on the spatial allocation of medical resources in coastal cities. Some scholars combined research areas by the comparative analysis of parameters of the accessibility model, and selected more suitable indicators for the spatial difference analysis of accessibility [7,8]. As the largest developing country, China has significant regional differences in accessibility to medical facilities, and influencing factors of accessibility in different regions are different [9]. Rapid urbanization has concentrated most of highly specialized medical facilities in built-up urban areas, while health institutions distributed in broad township areas can only offer basic medical services [10], and residents’ pursuit of highly specialized medical facilities leads to strong competition for medical resources. In order to solve the social impact of such problems, the government introduced a “graded diagnosis and treatment” policy that divides medical institutions according to the level of medical services (mainly divided into primary medical facilities that can undertake basic treatment services, and secondary and tertiary medical facilities that undertake highly specialized medical services) and guides residents’ medical behavior according to the level of medical facilities. Scholars have been focusing on accessibility to medical resources in Shaanxi under this policy in recent years, and proved that spatial difference in accessibility is still obvious [11,12]. However, there are differences between residents’ medical treatment mode and policy rules in reality. The existing research directly corresponds to residents’ medical-treatment mode with the level of medical institutions, which leads to the neglect of the primary-service ability of high-level medical institutions, and the different medical needs of residents impacting their medical behavior. There are few studies that evaluated accessibility of medical services according to the actual needs of residents, and analyzed differences in accessibility to medical services at different levels [13]. More importantly, current research tends to ignore the connotations of the accessibility model, so a single model is used for analyzing accessibility to medical services, which leads to a certain deviation in research results. Therefore, it is of great value to establish a model of actual medical behavior and study accessibility to medical services at different levels on the basis of residents’ actual medical needs.

At present, the research hotspot of medical-facility allocation has shifted from single accessibility analysis to spatial patterns of accessibility combined with equity evaluation. Early research ignored the social differentiation and deprivation effects among different groups [14], homogeneously treated all groups in a study area, and explored the spatial equity of all groups’ accessibility to medical facilities. In recent years, scholars have paid increasing attention to vulnerable groups, which are at a disadvantage when acquiring social wealth due to certain economic-development reasons. Accessibility to medical resources is also better among people with strong mobility and good economic conditions. Related research revealed that there are differences in accessibility to medical resources among different groups, and vulnerable groups have a deprivation effect when accessing medical resources [15,16,17,18,19,20,21]. However, Chinese scholars have not reached a consensus on the concept of equity among different groups, and there is a lack of relevant discussions. With the increasing differentiation of urban space and society, targeted research gradually emerges. Scholars found that low-income groups have a significant gap in community-supporting services compared with middle-high income groups [22], and household-registration populations have better accessibility to medical facilities than floating populations [3]. These conclusions reflect the necessity of studying differences in accessibility to medical resources for vulnerable groups in China. Especially at present, differences in the equity of different groups in obtaining three-level medical services on the basis of actual medical behavior are still unclear, and related issues urgently need to be explored.

As an important gateway connecting China’s western regions with the central and eastern provinces, the contradiction between supply and demand in Shaanxi province is more prominent than that in developed coastal areas of China. Since Shaanxi is an important public health-hub in western China, its accessibility evaluation can help guide the development of public health in other inland areas of the world. In view of this, this article takes Shaanxi, China as the research area, combines the distribution of medical-facility and census data on the grid scale, and from the perspective of time and space, and focuses on analyzing accessibility to three-level medical services and equity of accessibility in different areas and groups, with the view to provide a theoretical basis for relevant departments to make more accurate decisions on issues of public medical resources, and ensure public medical facilities are rationally and fairly allocated in the geographical space.

## 2. Materials

### 2.1. Research Area

Shaanxi province is located in the hinterland of inland China. It is composed of plateaus, mountains, plains, and other landforms. Terrain is high in the north and south, and low in the middle. From north to south, it forms the three geographical divisions of Shaanbei, Guanzhong, and Shaannan, with a total area of 205,600 km^2^. It has 10 cities and 107 county-level administrative units under its jurisdiction. In 2018, the permanent population was 38.644 million, and it is also the starting point of the Silk Road to Europe.

Shaanxi’s health industry has rapidly developed in recent years. The provincial government attaches great importance to the construction and reform of the public-health system. According to the 2018 Healthcare Development Statistical Bulletin, the province has a total of 35,300 medical and health institutions, 410,735 medical and health practitioners, and 253,711 hospital beds, which plays a supporting role in the development of medical and health services in the entire western region in China. Therefore, it was representative to select Shaanxi as the research area, where conclusions would be helpful in providing reference for other parts of the world, especially inland and economically underdeveloped areas.

### 2.2. Data Collection

Administrative-division data of basic spatial data required in this article came from the vectorization of the map of Shaanxi in the standard map service website of the Shaanxi Surveying and Mapping Geographic Information Bureau. Shaanxi road-network data were derived from 1:1,000,000 vector road data released by the National Geographic Information Bureau, which extracted various roads and railways of 2015. According to national-road safety regulations and the actual situation, the average speed of different types of roads and railways are as follows: national roads, 80 km/h; provincial roads, 70 km/h; county roads, 50 km/h; township roads, 25 km/h; special roads, 20 km/h; village roads, 15 km/h; high-speed and ordinary railways, 200 and 120 km/h, respectively; and the traffic network data sets (with and without railways) were constructed through the ArcGIS 10.1 platform. The grid data used the fishnet tool in the ArcGIS 10.1 platform (ESRI, Redlands, CA, USA) to divide Shaanxi into 8605 grids of 5 km × 5 km. The grid attribute was the number of people in the area, which was obtained by superimposing the population spatial data of the Chinese Academy of Sciences Resources and Environmental Data Cloud Platform. The point data of medical facilities were obtained through vectorization and spatial correction after crawling Baidu map data on the basis of Python programming.

**Figure 1:**
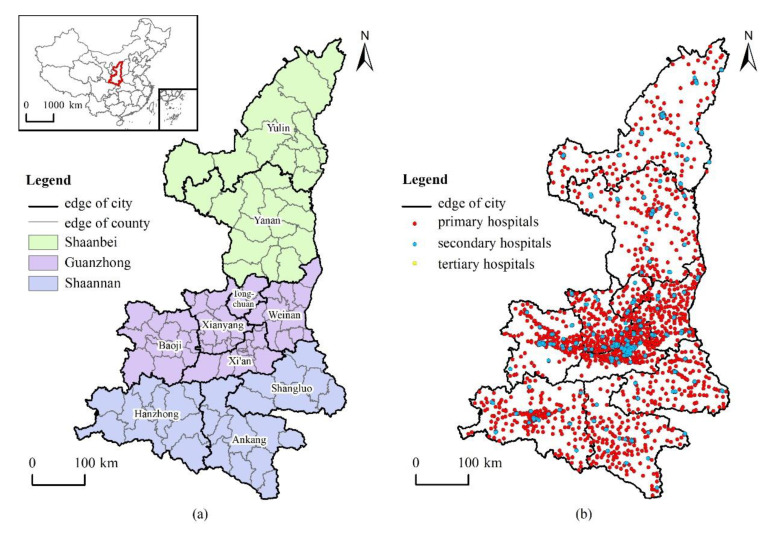
Study-area overview. (**a**) Shaanxi province; (**b**) public medical facilities.

When choosing medical institutions, the majority of the 35,300 medical institutions in Shaanxi are micro-institutions such as clinics, and their ability to provide medical services is limited. According to the classification of medical resources in the China Health and Family Planning Statistical Yearbook, we selected 1129 hospitals and professional public-health institutions, and 1432 basic medical and health institutions. The total number of medical institutions that provided medical services is 2561 (Figure 1), including general hospitals, traditional Chinese medicine hospitals, integrated Chinese and Western medicine hospitals, specialty hospitals, disease prevention and control centers, maternal and child health centers, community health service centers, township health centers, and street health centers. The service level of medical institutions is expressed by the number of employees, which was obtained from the official website of each medical institution, and different weights were given through their medical grade. Other data included socioeconomic, aging-population, and agricultural-population data of various regions that were obtained through the sixth national census and the Shaanxi Statistical Yearbook.

## 3. Methodology

### 3.1. Improved Network Analysis Model and Accessibility Calculation

**Figure 2:**
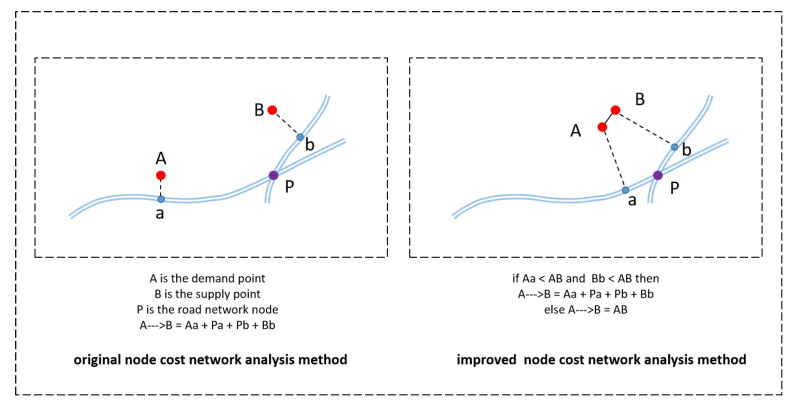
Improved node cost network analysis model diagram.

This article used an improved version of node cost network analysis method (Figure 2) [23]. Since spatial nodes in the study area did not completely fall on roads, the node cost network analysis method incorporates space-time distance cost from the starting and ending points to the roads into network analysis. However, considering that when the road network in the province was digitized, some roads were deleted or omitted because of their low level, the distance between starting and ending nodes is closer, but nodes are far from the road, and outliers were generated during the final network analysis. Therefore, this article set several conditions for the node cost network analysis method to improve it: When the cost of directly connecting the start and end nodes on the road network was less than the cost from the starting or ending nodes to the road network, the distance between starting and ending nodes used Euclidean distance as the network analysis result; otherwise, the result of node cost network analysis was used. When the distance was greater than threshold d, the time cost calculation used the road speed of 25 km/h; otherwise, the walking speed of 5 km/h was used. The choice of threshold d refers to other studies [11,20,24], and it was set as the distance that residents can walk in 15 min (1250 m).

**Figure 3:**
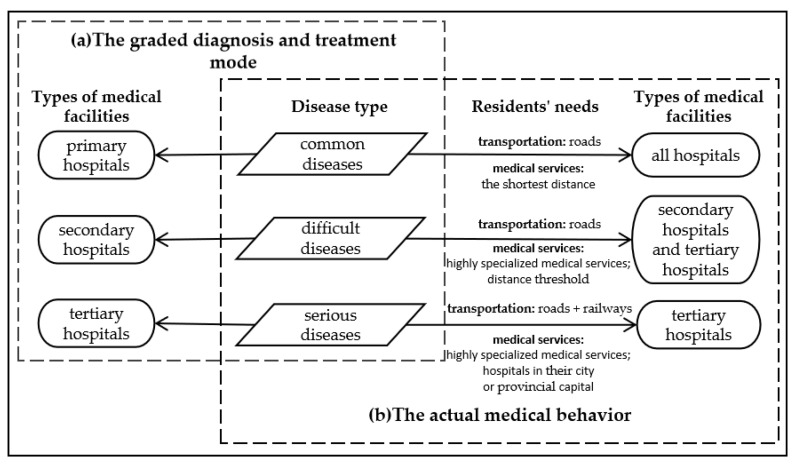
Concept map of (**a**) the graded diagnosis and treatment mode; (**b**) the actual medical behavior.

Different from the “graded diagnosis and treatment” model advocated by the government, medical-service levels were divided according to the actual medical demands of residents in Shaanxi (Figure 3). Residents have different medical needs and medical behaviors because of different types of diseases. Related studies divided diseases into common, difficult, and serious diseases according to the severity [25], and there are differences in medical-treatment patterns among residents under different disease types. In the case of common diseases, residents pay more attention to how to obtain medical resources with less space-time cost, but have weak requirements on the service level of medical facilities. Therefore, we selected all medical institutions in the province to provide primary medical services. With regard to difficult diseases, residents pay attention to the space-time cost to obtain medical services, and the service level and competition intensity of medical facilities. So, secondary general, traditional Chinese medicine, integrated Chinese and Western medicine, specialized hospitals and tertiary hospitals were selected for providing secondary medical services (376). With regard to serious diseases, residents are more concerned about the service level of medical institutions and have weak requirements for the space-time cost of obtaining medical resources. They usually choose tertiary hospitals in their city or provincial capital city for medical services, and the railway is also considered for transportation mode. In this article, 87 tertiary medical institutions were selected for tertiary medical services.

On this basis, the cost of obtaining medical resources was divided into time cost and spatial cost. Time cost changes with the improvement in transportation mode, and describes the difference in relative accessibility when the improvement in transportation produces space-time convergence. Spatial cost is an absolute cost that can describe the difference in absolute accessibility when there is lack of transportation.

#### 3.1.1. Space-Time Distance Model

Considering that residents’ demand for primary medical services is mainly concerned with space-time cost, so the nearest-distance method was used to calculate accessibility for residents in Shaanxi [26,27]. This method belongs to the space-time distance model, which calculates the space-time cost from the demand point of the research unit to the nearest medical-resource supply point. The formula is

(1) $$\mathrm{Pri}\_T_{i}/\mathrm{Pri}\_S_{i}=\min\left({\mathrm{Cos}t}_{ik}\right)$$

where Pri_T_*i*_/Pri_S_*i*_ represents minimal space-time cost Cost*_ik_* from demand point *i* to surrounding medical facility *k*; the smaller numerical value of Pri_T_*i*_/Pri_S_*i*_ was, the shorter distance for residents to obtain medical services was, and the better accessibility was.

#### 3.1.2. Two-Step Floating Catchment Area Method

The two-step floating catchment area (2SFCA) method can consider the scale of supply and demand points, reflect level of medical service and intensity of medical competition. It was used to calculate accessibility to secondary and tertiary medical services for residents in Shaanxi, but there were differences in the calculation methods of accessibility under different levels of medical service because of residents’ different needs. When residents obtained secondary medical services:

In the first step, for each medical facility *j*, we searched for demand point *k* within space-time threshold *d*_0_ of *j*, and used the power function as the distance-decay function to assign weights to the demand points and calculate supply-and-demand ratio R*_j_*:

(2) $$R_{j}=\frac{S_{j}P_{j}}{\sum_{k\in \left\{d_{kj}<d_{0}\right\}}D_{k}W_{kj}}$$

where *S_j_* represents the service ability of medical facilities expressed by the number of employees. *P_j_* is the weight of medical-service capacity. Considering that the medical service-level of the third-level hospital was relatively higher than that of others, its medical-service ability weight was set to 2, and the hospital service ability weight of the others was set to 1. *D_k_* is the population at the demand point, *W_kj_* is an attenuation function $f\left(d_{kj}\right)=d_{kj}^{-\beta }$ considering the distance-decay, which assumes that even in the same spatial scope, residents are more likely to obtain medical services closer to themselves. The value of β was between 1 and 2. According to results of other papers, we considered the impact of the attractiveness of secondary medical service to be greater than the effect of distance [28,29], and distance attenuation is low, so the value of β was set to 1; *d*_0_ is the space-time threshold. Because the study area is a provincial area with a large spatial range, it is more difficult for residents in remote areas to obtain medical resources than it is for residents in urban areas. So, the space-time threshold range had to be appropriately expanded to ensure that the model results were more accurate. Time threshold was set to 2 h with reference to other research results [30,31], and the corresponding spatial threshold was 10 km which is a 2 h walking distance.

In the second step, for each demand point *i*, we searched for all medical-resource supply points *j* within space-time threshold *d*_0_ of *i*. Supply-and-demand ratio *R_j_* of all *j* was weighted and summed through the distance-decay function. Lastly, the accessibility numerical-value of point *i* was obtained via

(3) $$\mathrm{Sec}\_T_{i}/\mathrm{Sec}\_S_{i}=\underset{j\in \{d_{ij}<d_{0}\}}{\sum }R_{j}W_{ij}=\underset{j\in \{d_{ij}<d_{0}\}}{\sum }\left\{\frac{S_{j}P_{j}}{\sum_{k\in \left\{d_{kj}<d_{0}\right\}}D_{k}W_{kj}}\right\}W_{ij}$$

where Sec_T_*i*_ and Sec_S_*i*_ represents the accessibility to secondary medical services under the threshold of time- and space-distance, respectively, its essence indicates the capacity level of medical service owned by residents at point *i*. The larger numerical value of accessibility was, the better level of accessibility to secondary medical services was, and vice versa.

When residents obtained tertiary medical services, the choice of medical institutions was tertiary hospitals in their or provincial capital city, and accessibility was the sum of accessibility to tertiary hospitals in their city and provincial capital city:

(4) $$A_{i}\_h=\underset{j\in j\_h}{\sum }\left\{\frac{S_{j}P_{j}}{\sum_{k\in k\_h}D_{k}W_{kj}}\right\}W_{ij}$$

(5) $$A_{i}\_p=\underset{j\in j\_p}{\sum }\left\{\frac{S_{j}P_{j}}{\sum_{k}D_{k}W_{kj}}\right\}W_{ij}$$

(6) $$\mathrm{Ter}\_T_{i}/\mathrm{Ter}\_S_{i}=A_{i}\_h+A_{i}\_p$$

where Ter_T_*i*_ and Ter_S_*i*_ represents the accessibility to tertiary medical services under the time- and space-distance, respectively. *A_i__h* and *A_i__p* indicate the accessibility of residents in their city and provincial capital city, respectively, *j_h* represents the tertiary hospitals in the city where the resident is located, and *j_p* represents the tertiary hospital in the provincial capital city. In Formula (4), the service scope of *j_h* is the city where the demand point *k_h* is located. In Formula (5), the service scope of medical institutions in provincial capital city is all the province. Considering that accessibility is less affected by space-time cost for serious diseases and residents tend to go to tertiary hospitals in the provincial capital city, the weight of service capacity of hospitals in provincial capital was set as 2, while that of hospitals in their city was 1. The railway was added to the traffic-network dataset, friction coefficients of *W_ij_* $\beta_{1}$ and $\beta_{2}$ were taken as 1.5/1 when residents went to hospitals in their city/provincial capital city, respectively. The higher numerical value of Ter_T_*i*_ /Ter_S_*i*_ was, the higher level of accessibility was, and vice versa.

### 3.2. Spatial Autocorrelation

Spatial autocorrelation comprises global and local spatial autocorrelation [32]. The former was used to analyze the spatial patterns of accessibility to three-level medical services in Shaanxi, which was usually expressed by Moran’s I index; the latter was used to analyze the internal structure of global spatial autocorrelation and the inter-relationship of local spaces of accessibility to three-level medical services in Shaanxi [33], which was usually expressed in LISA spatial cluster diagrams. The global spatial autocorrelation formula is

(7) $$I=\frac{n\sum_{i=1}^{n}\sum_{j\neq 1}^{n}W_{ij}\left(X_{i}-\bar{X}\right)\left(X_{j}-\bar{X}\right)}{\sum_{i=1}^{n}\sum_{j\neq 1}^{n}W_{ij}\sum_{i=1}^{n}{\left(X_{i}-\bar{X}\right)}^{2}}$$

where I is Moran’s I index, *W_ij_* is the spatial weight matrix, *n* is the number of research units, *X_i_* and *X_j_* are accessibility to three-level medical services in units *i* and *j*, respectively, and $\bar{X}$ is the average of accessibility of all units. Moran’s I index is in the range of [−1,1]. When I is less than 0, it indicates a negative spatial correlation, i.e., discrete distribution; when I is greater than 0, it indicates positive spatial correlation, which means agglomerated distribution. When I equals 0, the study object is randomly distributed.

The local spatial autocorrelation formula is

(8) $$I_{i}=Z_{i}\overset{n}{\underset{j\neq 1}{\sum }}W_{ij}Z_{j}$$

where I_*i*_ is local spatial autocorrelation Moran’s I index, indicating the degree of influence of the county unit *i* by *j*; and Z_*i*_ and *Z_j_* are standardized values of accessibility to three-level medical services of *X_i_* and *X_j_*, respectively.

### 3.3. Lorenz Curves and Gini Coefficient

The Gini coefficient is a commonly used indicator in economics to measure the income gap of residents in a country or region that has been applied to various disciplines. It is often used to describe the spatial distribution of discrete areas in geography, and can also be used to compare spatial-distribution differences of geographical elements [34]. We used it to evaluate equity of accessibility to three-level medical services in Shaanxi.

The Gini coefficient can be calculated with the Lorenz curve, which is a visual expression of equity of accessibility to three-level medical services in Shaanxi. The horizontal axis represents population accumulation, and the vertical axis represents accessibility accumulation. The diagonal line connecting the two ends of the curve is an equal-distribution line [35,36]. The Gini coefficient is the ratio of the area between the equal-distribution line and the Lorenz curve to the area of all regions under the equal-distribution line. The greater spatial differences in accessibility was, the larger value of Gini coefficient was.

## 4. Results

### 4.1. Spatial Difference in Accessibility to Three-Level Medical Services

#### 4.1.1. Spatial Difference in Accessibility to Primary Medical Services

**Figure 4:**
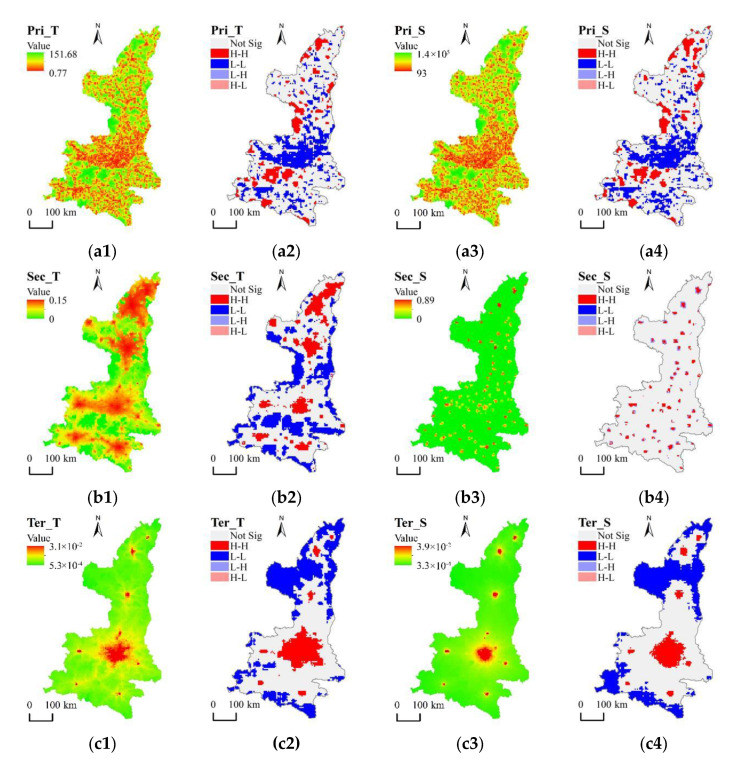
Accessibility to (**a1**,**a3**) primary medical services (Pri_T, Pri_S); (**b1**,**b3**) secondary medical services (Sec_T, Sec_S); (**c1**,**c3**) tertiary medical services (Ter_T, Ter_S); and LISA spatial cluster of (**a2**,**a4**) Pri_T and Pri_S; (**b2**,**b4**) Sec_T and Sec_S; (**c2**,**c4**) Ter_T and Ter_S in Shaanxi. Notes: Red areas represent high level of accessibility to three-level medical services and green areas represent low level of accessibility in (**a1**,**a3**,**b1**,**b3**,**c1**,**c3**).

The calculation results of accessibility to primary medical services for residents in Shaanxi are shown in Figure 4.

The overall spatial pattern of accessibility to primary medical services is balanced in Shaanxi. Areas with high level of accessibility have a tendency to distribute along the road network and form partitions in the Qinling mountains, connecting to Shaannan through the corridor. Guanzhong generally has a high level of accessibility, and the average cost of minimal time distance is just 18.88 min. The time cost for residents to obtain primary medical services is significantly better than that in Shaannan and Shaanbei. At the same time, Moran’s I shows that Guanzhong has the highest agglomeration degree of accessibility. Although the agglomeration degree of accessibility is low, the overall level of accessibility is poor and in a weakly balanced state in Shaanbei.

Spatial-correlation patterns of accessibility in Shaanxi and three major subdivisions presented significant spatial agglomeration. The LISA spatial cluster diagrams (Figure 4(a2,a4)) showed that Pri_T in Guanzhong is of the low-low agglomeration type, which represents a cluster with high level of accessibility that the distance to primary medical services is short in the areas, with Baoji-Xi’an-Weinan generally as the axis. The spatial-correlation patterns of accessibility in Shaanbei and Shaannan have obvious regional characteristics. The low–low agglomeration type in the built-up area of each county is obvious, while remote towns, and rural and mountainous areas have characteristics of high–high agglomeration, indicating that the overall accessibility level in these areas is poor, so residents need to pay more space-time costs to obtain primary medical services.

#### 4.1.2. Spatial Difference in Accessibility to Secondary Medical Services

Figure 4(b1,b3) show the spatial patterns of the time- and the spatial-distance threshold of accessibility, respectively, to secondary medical services (Sec_T, Sec_S) for residents in Shaanxi. The spatial pattern of Sec_T takes the built-up areas of each city as the core and decreases outward along the road network. High-level corridors of accessibility were formed between cities, and the level of accessibility of urban areas is generally higher than that of counties under their jurisdiction, which formed a banded multicore spatial structure. Moran’s I showed that Guanzhong has a small agglomeration degree of accessibility to secondary medical services. Although the level of Sec_T is good in Shaanbei, there is a large agglomeration degree of Sec_T, and most remote rural areas have a significant gap in accessibility to secondary medical services compared with urban areas. The overall level of Sec_S in various regions is obviously lower than Sec_T, especially in vast rural areas.

Spatial-correlation patterns of accessibility in various regions exhibited spatial agglomeration characteristics (Table 1), but the aggregation degree of Sec_S was relatively low and the aggregation range is small. The reason is that traffic conditions restrict the travel range of residents, lowering the accessibility of many regions in the province, and resulting in a decrease in the level of agglomeration.

#### 4.1.3. Spatial Difference in Accessibility to Tertiary Medical Services

Figure 4(c1,c3) show the spatial patterns of time- and spatial-distance accessibility, respectively, to tertiary medical services (Ter_T, Ter_S). Ter_T takes the built-up area of each city as the high-level center of accessibility, and forms a banded multicore spatial pattern along the road network. Xi’an has obvious location advantages, and areas with high-level accessibility are distributed in blocks. The overall core-edge structure of accessibility in Shaanxi is prominent, as each city takes Xi’an as the main core and forms a subcore. Driven by Xi’an, the accessibility level of Guanzhong is good (Table 1), but spatial agglomeration is significant; Shaannan and Shaanbei occupy a vast area, which leads to inconvenient transportation and poor accessibility, but the degree of spatial aggregation is small.

Due to the concentration of tertiary medical institutions, spatial agglomeration characteristics of accessibility are obvious and the degree of agglomeration is high. Figure 4(c2,c4) show that accessibility in the built-up areas of each city presents a high-high agglomeration, indicating that the medical-service capacity in urban areas is large, and the level of accessibility is high in the areas, and Xi’an has the largest scope of agglomeration, while low-low clustering characteristics are obvious in large villages and other areas far away from the urban built-up areas.

By comparing spatial differences in accessibility patterns to three-level medical services, the accessibility level of primary medical services was found to be good, and spatial distribution was balanced; areas with high-level accessibility are scattered along the road network, and residents can obtain primary medical services with fewer space-time costs. Accessibility to secondary medical services has a banded and multicore spatial distribution pattern; areas with high-level accessibility are clustered in urban built-up areas. The Sec_S spatial pattern takes county-level administrative districts as the core to form high-level accessibility-isolated points, which shows that the difference between urban and rural areas is quite large. The core-edge feature of accessibility to tertiary medical services is prominent, which forms a banded multicore spatial structure with Xi’an as the main core and other cities as subcores. The differences in accessibility among different regions are further expanded, which is more obvious in the LISA spatial cluster diagram. The high-high agglomeration type is mainly formed in the urban built-up areas of Xi’an, and the accessibility level of other areas was obviously weaker than that of Xi’an.

Observing different regions, accessibility to three-level medical services in Guanzhong was found to be good. Because of its location in the Qinling mountains, residents in Shaannan need to pay more time and spatial costs to obtain medical resources compared with those in Guanzhong. Accessibility to primary medical services for residents in Shaanbei is poor, where accessibility to secondary medical services is good because competition pressure for highly specialized medical facilities is small.

### 4.2. Equity in Obtaining Medical Services Based on Accessibility

#### 4.2.1. Equity of Accessibility to Medical Services in Different Areas

Equity analysis of accessibility to medical services is mainly to explore whether accessibility is evenly distributed to each individual in different areas and whether there is spatial deprivation in Shaanxi.

The Gini coefficient fully considers differences in the number of populations in different regions, and evaluates equity of accessibility by taking individuals as the research object. Since fewer space-time costs in the space-time distance model represents a high level of accessibility, in order to make high numerical-value of accessibility correspond to high level of accessibility to primary medical services, we took the reciprocal of Pri_T/Pri_S and performed a min–max normalization process. The new results of accessibility were accumulated with the population data of each region to draw the Lorenz curve and calculate the Gini coefficient.

**Figure 5:**
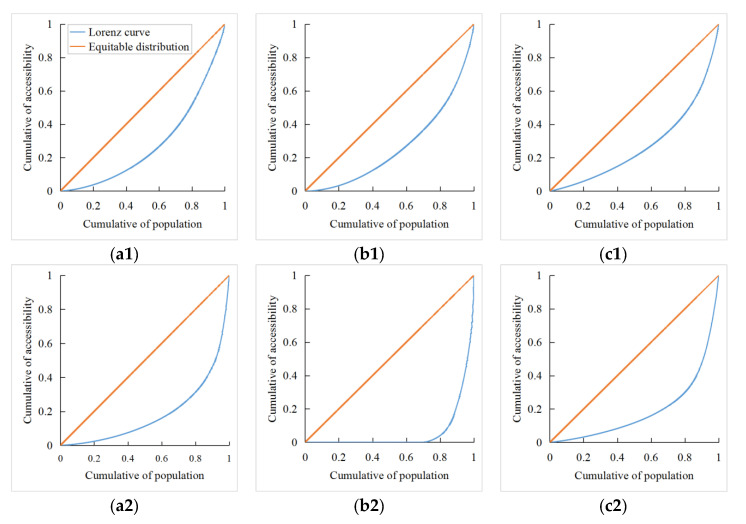
Lorenz curve of accessibility to (**a1**,**a2**) primary medical service (Pri_T, Pri_S); (**b1**,**b2**) secondary medical service (Sec_T, Sec_S); (**c1**,**c2**) tertiary medical service (Ter_T, Ter_S) in Shaanxi.

Figure 5 are the Lorenz curves of accessibility to three-level medical services for residents in Shaanxi. The curvature of Lorenz curves of accessibility to secondary and tertiary medical services was greater than that of accessibility to primary medical services. Due to the distance threshold of accessibility to secondary medical services, the curvature of the Lorenz curve was large, and spatial inequality was obvious. The curvature of Lorenz curves of Pri_S/Sec_S/Ter_S was greater than that of Pri_T/Sec_T/Ter_T. The Gini coefficients of three major geographic subdivisions were respectively calculated with the Lorenz curves (Table 2).

The spatial inequality of accessibility to three-level medical services was prominent. The Gini coefficients of accessibility is high in most areas. At the same time, due to the remote location of Shaannan and Shaanbei, residents in most areas had poor accessibility to tertiary medical services, which produced a low-level balanced state, and lead to a low Gini coefficient of accessibility. Equity of accessibility to primary medical services was generally better than that to secondary and tertiary medical services, which is mainly due to the concentrated distribution of highly specialized medical facilities in urban built-up areas, and the spatial deprivation for residents to obtain highly specialized medical service was obvious.

Equity of accessibility to secondary and tertiary medical services in Guanzhong was better than that in other regions, and level of accessibility to three-level medical services was generally good, so it was in a strong-balance state relative to other regions. Affected by the Loess plateau and Qinling mountains, Shaanbei and Shaannan, separately, have inconvenient transportation systems, social resources are highly concentrated in urban built-up areas, and a large number of nonurban populations have difficulty in obtaining medical resources. Due to the superior socioeconomic conditions and great development potential in Xi’an, the provincial capital, the radiation effect drives the development of surrounding areas in Guanzhong. Therefore, levels of accessibility and equity are both generally good.

The Gini coefficients of spatial-distance accessibility were generally larger than those of time-distance accessibility; the Gini coefficient of Sec_S was close to 1. Since Pri_S, Sec_S and Ter_S represent the absolute accessibility of residents to obtain three-level medical services, when there is a lack of transportation, residents in different regions have obvious difference in accessibility.

#### 4.2.2. Equity of Accessibility to Medical Services in Different Groups

In order to study whether vulnerable groups had a deprivation effect in access to medical services, The aging-population (over 65 years old) and agricultural-population data were separately selected, and their correlation with accessibility of various counties was analyzed in order to explore the equity of accessibility to medical services for different groups in Shaanxi. The accessibility of each county is obtained by weighting the accessibility numerical-value of the grid points in the administrative division with its population.

**Figure 6:**
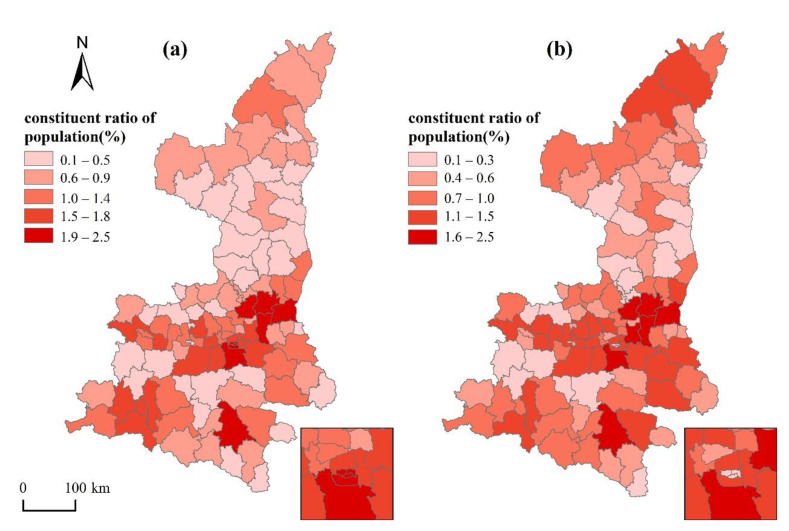
Spatial distribution of (**a**) aging and (**b**) agricultural populations.

Figure 6 shows that the spatial distribution of aging population and agricultural population in Shaanxi. The spatial distribution of the agricultural population is relatively uniform, and aging populations gather in counties with better socioeconomic conditions, especially in the urban built-up areas of Xi’an, where the aging population is large and dense, and the agricultural population is less distributed.

In order to study the relationship between accessibility and group distribution, Pearson correlation analysis was performed between population-composition data and county accessibility (Table 3). On the provincial scale, there was no obvious correlation between aging-population ratio and accessibility, while agricultural–population ratio was significantly related to accessibility. The higher agricultural–population ratio was, the lower level of accessibility to three-level medical services was.

From the perspective of the three major geographic subdivisions, accessibility to three-level medical services for the agricultural population in Guanzhong had a deprivation effect. However, there was no significant deprivation effect of accessibility to primary medical services for the aging population; the higher aging population ratio was, the higher level of accessibility to secondary and tertiary medical services was. There was no obvious deprivation effect of accessibility to primary medical services for aging and agricultural populations in Shaannan, but agricultural populations are widely distributed in areas with poor accessibility to secondary and tertiary medical services, leading to significant inequality. The agricultural population in Shaanbei has been deprived of accessibility to tertiary medical services, and the aging and the agricultural populations had a deprivation effect under spatial-distance accessibility to secondary medical services, but there was no significant unfair phenomenon under time-distance accessibility.

**Figure 7:**
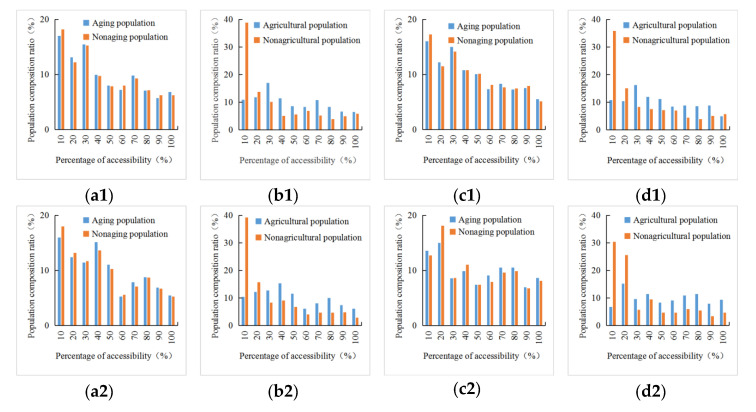

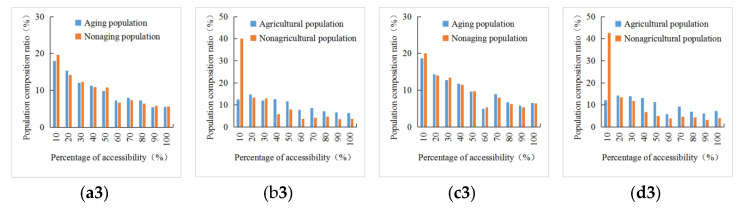
Differences of (**a1**) Pri_T; (**a2**) Sec_T; (**a3**) Ter_T; (**c1**) Pri_S; (**c2**) Sec_S; (**c3**) Ter_S between aging and nonaging populations, and (**b1**) Pri_T; (**b2**) Sec_T; (**b3**) Ter_T; (**d1**) Pri_S; (**d2**) Sec_S; (**d3**) Ter_S between agricultural and nonagricultural populations in Shaanxi.

Figure 7(a1–a3,c1–c3) are the composition ratio of aging and nonaging populations with different percentiles of Pri_T, Sec_T, Ter_T, Pri_S, Sec_S, Ter_S levels, respectively. No matter how accessibility to three-level medical services changed, there was no significant difference in composition ratio with different percentiles of accessibility levels. The main reason was that the aging population in Shaanxi was more distributed in regions with developed economy and higher accessibility. Therefore, there was no obvious deprivation effect for the aging populations to obtain medical services compared with nonaging populations.

Similarly, Figure 7(b1–b3,d1–d3) are the composition ratio of the agricultural and nonagricultural populations with different percentiles of Pri_T, Sec_T, Ter_T, Pri_S, Sec_S, Ter_S levels, respectively. Under different accessibility quantiles, the agricultural and nonagricultural population was quite different. Due to different social divisions of labor, the agricultural population was more distributed in remote areas with lower accessibility levels. Spatial isolation led to a significantly lower agricultural-population composition ratio than that of the nonagricultural population in high-accessibility areas, and ultimately resulted in group differences, which is more evident in the top 10% quantile of accessibility. Therefore, different vulnerable groups had differences in their ability to obtain medical resources. In order to improve the equity of accessibility, policy makers should specifically consider the situation of various groups in different regions.

### 4.3. Sensitivity Analysis of Service Threshold and Service-Capability Weight Setting

Sensitivity analysis of accessibility was carried out with the service threshold of 30 min as the interval, and service-capacity weight of 0.5 as the interval; statistical results are shown in Table 4. At different service thresholds, accessibility to medical services had an increasing trend with the expansion of the threshold range. The top 80% quantile of accessibility showed that the larger the threshold was, the higher level of accessibility. Since medical service-capacity is fixed, there was a certain decrease in the high-accessibility part. Meanwhile, under different service-capability weights, the statistics of accessibility increase with the increase in weights.

**Figure 8:**
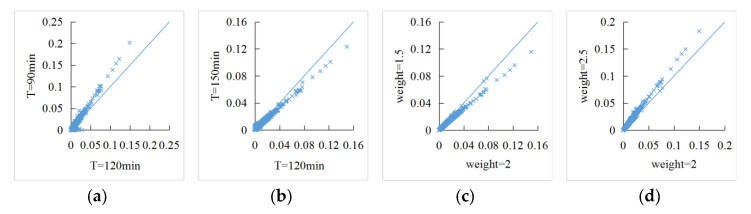
Comparisons of scatter plots of accessibility with different (**a**,**b**) service thresholds (T = 90 min, T = 150 min); (**c**,**d**) service-capacity weights (W = 1.5, W = 2.5).

Figure 8a,b are comparisons of scatter plots of accessibility with different service thresholds. Accessibility numerical-values were generally distributed along the 45 degree line, and threshold selection did not cause the accessibility numerical-value to obviously change. Similarly, Figure 8c,d are comparisons of scatter plots of accessibility with different service-capacity weights. The change of weights made accessibility linearly change, but the range was small, and the overall accessibility numerical-value was still distributed around the 45 degree line.

## 5. Discussion

### 5.1. Improvement in Traffic Conditions Can Reduce Difference in Accessibility to Medical Services

An improvement in traffic conditions obviously leads to an improvement in accessibility. Many scholars confirmed that an improvement in traffic conditions can produce space-time convergence and cause the Matthew effect to expand imbalance or cause the trickle-down effect to weaken imbalance between regions [37,38]. Considering such studies are rare in the field of public service facilities, scholars pay more attention to the difference in accessibility to public service facilities under different travel methods [39]. This article compares the absolute accessibility (Pri_S/Sec_S/Ter_S) and relative accessibility (Pri_T/Sec_T/Ter_T) of residents in Shaanxi to explore the impact of traffic conditions on accessibility to three-level medical services.

First, comparing Pri_T and Pri_S, although traffic improvement produces space-time convergence, it generally has limited impact on the spatial pattern of accessibility to primary medical services. Therefore, regional differences in accessibility have not significantly changed. However, the improvement in traffic conditions has large impact on the accessibility to secondary and tertiary medical services, especially in rural areas. Compared with Sec_T, the LISA spatial cluster diagram of Sec_S presented a pointlike high-high agglomeration-type block, while its periphery produced a low-high aggregation-type ring envelope, indicating that accessibility to secondary medical services has significant regional differences, and the improvement in traffic conditions can enhance the regional spatial balance through the trickle-down effect.

Meanwhile, the Gini coefficients of spatial-distance accessibility were generally larger than those of time-distance accessibility, and the gap between Gini coefficients of Sec_T and Sec_S was especially large. Therefore, transportation mode has significant impact on equity of accessibility, especially high-level medical services. The improvement in transportation mode can significantly weaken spatial deprivation. Comparing the correlation coefficient of Pri_S/Sec_S/Ter_S in each major geographical subdivision with that of Pri_S/Sec_S/Ter_S, the correlation coefficient of Pri_S/Sec_S/Ter_S was found to be small. In Shaanbei, the vulnerable groups have especially significant Sec_S inequity, but the correlation coefficient of Sec_T did not show deprivation effect, indicating that the improvement in traffic conditions reduces the cost of obtaining medical services for residents in remote areas and effectively alleviates group differences. Although transportation conditions in Guanzhong are good, which has weakened social inequity, the dense population and high pressure on competition for medical resources have produced a deprivation effect for some vulnerable groups. In general, developing public transportation is an effective means to improve equity of accessibility to medical services.

### 5.2. Improvement in Hospital Service Level Has No Significant Effect on Accessibility Spatial Pattern

Through the sensitivity analysis of service distance and service-capability weight setting, it can be seen that the expansion of the service distance of medical institutions will increase the level of accessibility to medical services in low-accessibility areas; while the increase in the service-capability weight of medical institutions can make overall numerical-value of accessibility linearly increases, and improved level of accessibility to medical services.

At the same time, changes in service-capability weight of medical institutions didn’t have a dramatic impact on the spatial pattern of accessibility to medical services. Although changes in scope of services of medical institutions had a certain impact on accessibility in different regions, there was no significant changes in numerical-value of accessibility. The change service-capacity weight of medical institutions can make the accessibility linearly change, but the overall change range was not large. Because of the linear change, the spatial pattern of accessibility did not have a dramatic impact. Therefore, improving the service level of medical institutions can effectively improve level of accessibility to medical service in all regions, especially remote areas with poor accessibility, and did not have a significant impact on spatial difference of accessibility.

### 5.3. Policy Suggestion

Through the above research, in order to improve the spatial balance of accessibility in Shaanxi and provide reference for the development of public health in other inland areas of the world, the following are suggested: (1) To solve the problem of insufficient medical resources, the government should promote investment, attract or encourage through policy more medical institutions and talents to flow into inland areas, and expand medical service-capacity, improving the level of accessibility to medical services, so as to alleviate the competition pressure of medical resources. (2) In order to solve the problem of polarization of medical-service accessibility and high cost of medical treatment for residents in remote areas, it is necessary to increase the construction of basic transportation networks, especially the road networks in mountainous and hilly areas like Shaannan and Shaanbei, and transfer financial investment from the construction of high-level and large-scale road networks to the improvement of low-level and small-scale road networks. The government should promote the trickle-down effect of central cities, guide the inflow of some medical resources to the underdeveloped areas, and evacuate agglomeration centers to achieve a balance among regions. (3) The government should plan and allocate medical resources in a unified way. At the same time, the needs of different social groups should be considered, and the policy of “positive differential treatment” needs to be implemented to help socially vulnerable groups overcome the difficulties of medical treatment.

## 6. Conclusions

We used the improved node cost network analysis method, the space-time distance model, and the two-step floating catchment area method to explore spatial differences in accessibility to three-level medical services on the basis of actual medical behavior in Shaanxi. The main conclusions are as follows:

(1) The overall level of accessibility to primary medical services in Shaanxi is good, and the spatial pattern of accessibility is balanced. Areas with high level of accessibility have a trend of distribution along the road network. Due to the superior traffic conditions in Guanzhong, the cost of obtaining primary medical services is lower than that in Shaanbei and Shaannan. The spatial-correlation pattern mainly presented as a low–low agglomeration type.

The spatial differences in accessibility to secondary and tertiary medical services were obvious in Shaanxi. With the built-up areas of various cities as the core, level of accessibility decreases outward along the road network. High-level accessibility corridors were formed between various cities, and the accessibility level of cities is higher than that of counties under their jurisdiction, indicating that the phenomenon of internal differentiation in cities is prominent.

(2) The spatial-deprivation effect of accessibility to secondary and tertiary medical services is more obvious than that to primary medical services. There are differences in the equity of accessibility in different regions. The equity of accessibility to primary and secondary medical services is good in Guanzhong, but equity of accessibility to three-level medical services is generally poor in other regions. The Gini coefficients of spatial-distance accessibility are larger than time-distance, indicating that if there is a lack of transportation, residents have serious spatial deprivation.

(3) Different vulnerable groups have differences in their ability to obtain medical services, and the evaluation results of the equity of accessibility were also different. There was no obvious inequity of accessibility between nonaging and aging populations in Shaanxi. However, the deprivation effect of accessibility for agricultural populations was more prominent than that for nonagricultural populations.

In addition, by exploring the impact of traffic conditions on accessibility, improved traffic conditions were found to have a significant impact on reducing regional differences and improving accessibility to secondary and tertiary medical services, especially in rural areas. At the same time, improved traffic conditions can produce space-time convergence, and alleviate the gap in the ability of different groups to obtain medical resources and promote equity among groups.

This article macroscopically analyzed the spatial differences and equity of accessibility to three-level medical services in Shaanxi. Restricted by data access, the impact on accessibility of small and micromedical facilities such as clinics was not considered. In addition, residents’ medical behavior is affected by multiple factors, such as subjective factors, economic capabilities, and referral treatment in the “graded diagnosis and treatment” model, which increase the complexity of the simulation of actual situations. In the future, the above factors need to be considered for indepth research.

## Figures and Tables

**Table 1:** Statistical analysis of accessibility.

**Region**	**Pri_T**				**Sec_T**				**Ter_T**			
	**Max**	**Min**	**Mean**	**Moran’ I**	**Max**	**Min**	**Mean**	**Moran’ I**	**Max**	**Min**	**Mean**	**Moran’ I**
Province	151.68	0.77	23.90	0.742	0.15	0	4.7 × 10^−3^	0.830	3.1 × 10^−2^	5.3 × 10^−4^	1.7 × 10^−3^	0.847
Guanzhong	132.13	1.12	18.88	0.762	0.05	0	4.8 × 10^−3^	0.819	2.1 × 10^−2^	9.1 × 10^−4^	2.5 × 10^−3^	0.913
Shaanbei	140.87	1.56	27.14	0.706	0.15	0	6.0 × 10^−3^	0.829	3.1 × 10^−2^	5.5 × 10^−4^	1.3 × 10^−3^	0.688
Shaannan	151.68	0.77	24.13	0.728	0.07	0	3.4 × 10^−3^	0.828	1.7 × 10^−2^	5.3 × 10^−4^	1.6 × 10^−3^	0.670
**Region**	**Pri_S**				**Sec_S**				**Ter_S**			
	**Max**	**Min**	**Mean**	**Moran’ I**	**Max**	**Min**	**Mean**	**Moran’ I**	**Max**	**Min**	**Mean**	**Moran’ I**
Province	1.4 × 10^5^	93	1.1 × 10^4^	0.757	0.89	0	3.9 × 10^−3^	0.530	3.9 × 10^−2^	3.3 × 10^−4^	1.2 × 10^−3^	0.768
Guanzhong	1.4 × 10^5^	93	8.6 × 10^3^	0.672	0.30	0	3.2 × 10^−3^	0.365	3.3 × 10^−2^	5.1 × 10^−4^	1.8 × 10^−3^	0.860
Shaanbei	6.4 × 10^4^	223	1.3 × 10^4^	0.767	0.89	0	4.9 × 10^−3^	0.467	3.9 × 10^−2^	3.3 × 10^−4^	0.8 × 10^−3^	0.680
Shaannan	7.9 × 10^4^	121	1.0 × 10^4^	0.764	0.48	0	3.1 × 10^−3^	0.563	3.5 × 10^−2^	4.0 × 10^−4^	1.0 × 10^−3^	0.576

**Table 2:** Gini coefficient of medical accessibility in different regions.

Region	Pri_T	Pri_S	Sec_T	Sec_S	Ter_T	Ter_S
Province	0.446	0.636	0.471	0.883	0.457	0.623
Guanzhong	0.392	0.613	0.436	0.816	0.414	0.577
Shaanbei	0.442	0.492	0.518	0.985	0.257	0.409
Shaannan	0.459	0.532	0.479	0.961	0.215	0.332

**Table 3:** Correlation analysis between population and accessibility.

Accessibility	Province		Shaannan	
	Aging Population	Agricultural Population	Aging Population	Agricultural Population
**Pri_T**	−0.102	0.327 **	0.199	0.241
**Pri_S**	−0.114	0.338 **	0.241	0.210
**Sec_T**	0.079	−0.754 **	−0.101	−0.754 **
**Sec_S**	0.131	−0.829 **	−0.218	−0.798 **
**Ter_T**	0.156	−0.747 **	−0.417 *	−0.558 **
**Ter_S**	0.164	−0.752 **	−0.510 *	−0.519 *
	**Guanzhong**		**Shaanbei**	
	**Aging Population**	**Agricultural Population**	**Aging Population**	**Agricultural Population**
**Pri_T**	−0.203	0.331 *	0.072	−0.282
**Pri_S**	−0.230	0.376 **	0.253	−0.161
**Sec_T**	0.398 **	−0.773 **	−0.339	−0.333
**Sec_S**	0.468 **	−0.854 **	−0.426 *	−0.582 **
**Ter_T**	0.339 *	−0.761 **	−0.306	−0.654 **
**Ter_S**	0.351 **	−0.762 **	−0.385	−0.662 **

Notes: ** *p* < 0.01 (two-tailed); * *p* < 0.05 (two-tailed).

**Table 4:** Sensitivity analysis.

Accessibility		Tolerance			Weight		
		T = 90	T = 120	T = 150	W = 1.5	W = 2	W = 2.5
**Percentile** **(%)**	10	0	1.6 × 10^−4^	5.3 × 10^−4^	1.6 × 10^−4^	1.6 × 10^−4^	1.6×10^−4^
	20	2.5 × 10^−4^	8.7 × 10^−4^	1.5 × 10^−3^	8.5 × 10^−4^	8.7 × 10^−4^	8.8×10^−4^
	30	9.1 × 10^−4^	1.6 × 10^−3^	2.4 × 10^−3^	1.5 × 10^−3^	1.6 × 10^−3^	1.6×10^−3^
	40	1.5 × 10^−3^	2.3 × 10^−3^	3.1 × 10^−3^	2.1 × 10^−3^	2.3 × 10^−3^	2.4 × 10^−3^
	50	2.0 × 10^−3^	3.1 × 10^−3^	3.7 × 10^−3^	2.8 × 10^−3^	3.1 × 10^−3^	3.5 × 10^−3^
	60	2.9 × 10^−3^	3.9 × 10^−3^	4.4 × 10^−3^	3.5 × 10^−3^	3.9 × 10^−3^	4.5 × 10^−3^
	70	4.1 × 10^−3^	5.0 × 10^−3^	5.1 × 10^−3^	4.4 × 10^−3^	5.0 × 10^−3^	5.8 × 10^−3^
	80	5.8 × 10^−3^	6.2 × 10^−3^	6.1 × 10^−3^	5.4 × 10^−3^	6.2 × 10^−3^	7.4 × 10^−3^
	90	8.5 × 10^−3^	8.1 × 10^−3^	7.6 × 10^−3^	7.0 × 10^−3^	8.1 × 10^−3^	9.6 × 10^−3^
	100	2.0 × 10^−2^	1.6 × 10^−2^	1.4 × 10^−2^	1.4 × 10^−2^	1.6 × 10^−2^	2.0 × 10^-2^
max		0.20	0.15	0.12	0.12	0.15	0.18
min		0	0	0	0	0	0
mid		2.4 × 10^−3^	3.5 × 10^−3^	4.0 × 10^−3^	3.2 × 10^−3^	3.5 × 10^−3^	4.0 × 10^−3^
mean		4.6 × 10^−3^	4.7 × 10^−3^	4.9 × 10^−3^	4.1 × 10^−3^	4.7 × 10^−3^	5.5 × 10^−3^
std		7.7 × 10^−3^	5.9 × 10^−3^	4.9 × 10^−3^	4.8 × 10^−3^	5.9 × 10^−3^	7.2 × 10^−3^

## Data Availability

Data available on request due to restrictions privacy. The data presented in this study are available on request from the corresponding author.
